# Temperature regulation of cell cycle and growth dynamics in *Arabidopsis*

**DOI:** 10.1042/BST20260121

**Published:** 2026-07-31

**Authors:** Vasundara Sundaravadivelu, Ritesh Kumar Raipuria, Aashish Ranjan

**Affiliations:** BRIC-National Institute of Plant Genome Research, Aruna Asaf Ali Marg, New Delhi 110067, India

**Keywords:** Arabidopsis thaliana, Cell cycle, Cold/chilling stress, Heat stress, High temperature, Plant growth and development

## Abstract

The plant cell cycle is a highly coordinated and regulated process that integrates endogenous and environmental signals to control cell division, meristem maintenance, and cell fate specification for growth and development. Temperature is a critical environmental signal that regulates the cell cycle to manifest developmental plasticity in *Arabidopsis* roots and shoots. *Arabidopsis* plants exhibit either adaptive growth responses or arrested growth, depending on the temperature regime. The temperature-mediated growth dynamics in *Arabidopsis* involve altered cell-cycle regulation. While plant developmental and physiological responses to temperature have been extensively studied, the integration of temperature signalling cues with cell-cycle dynamics to regulate growth adaptation remains poorly understood. The present review not only compiles existing information on temperature-mediated regulation of cell-cycle dynamics but also provides a perspective on multidisciplinary approaches to investigate cell-cycle dynamics at spatiotemporal resolution in *Arabidopsis* adaptive growth responses.

## Introduction

Plant growth and developmental responses, with significant underpinnings in cell-cycle control, are strongly integrated with ambient environmental conditions. Temperature is one of the most important environmental factors with a profound impact on plant growth dynamics, as it fluctuates both diurnally and seasonally. Temperature controls all major developmental and physiological processes, from seed germination to vegetative and reproductive development [1–3]. Plants exhibit extensive reprogramming of genetic and molecular mechanisms in response to changes in optimal temperature, leading to altered growth and developmental dynamics. For example, prolonged cold-induced silencing of flowering locus C, also called vernalisation, is required to promote flowering in many temperate crops [4]. In contrast, elevated temperatures accelerate flowering as PHYTOCHROME INTERACTING FACTOR 4 (PIF4) directly activates FLOWERING LOCUS T (FT) [5]. Depending on the temperature range and the adaptive responses, plant responses to temperature can be either acclimation or tolerance. Exposure to milder temperature changes, such as low temperature/chilling (4–15°C) and high ambient temperature (25–30°C) in *Arabidopsis*, elicits acclimation responses that confer adaptive mechanisms to alleviate the effects of sub-optimal conditions. In contrast, a tolerance response is elicited when plants are exposed to more severe temperatures, such as cold and heat stress, where plants prioritise survival over growth; hence, growth is generally attenuated or arrested [6–8]. For example, leaf area reduction and petiole elongation in response to high ambient temperature to aid in rosette cooling are acclimation responses. In contrast, heat-stress-induced arrest of root and shoot growth is a tolerance response. Together, optimisation of growth dynamics is integral to acclimation or tolerance responses to temperature changes. Plants modulate growth dynamics by integrating temperature- and phytohormone-signalling pathways with cell division, elongation, and differentiation.

Perception of ambient temperature is the first step in eliciting acclimation or a tolerance response to modulate cell cycle and growth dynamics. Several thermosensors, including membrane integrity/fluidity, photoreceptors such as Phytochrome B (PhyB), protein stability, RNA structure, and chromosome conformation, have been identified over the years [9–11]. Downstream of perception, low-temperature signalling is mediated by the INDUCER of CBF EXPRESSION 1 (ICE1)–C-REPEAT BINDING FACTOR (CBF) pathway in *Arabidopsis*. Cold-induced stabilisation of ICE1 promotes the expression of CBFs, which then activate multiple cold-responsive genes (CORs) [12,13]. COR genes aid in membrane stabilisation, osmoprotection, and metabolic shifts, which collectively enhance cold acclimation and/or tolerance. In contrast, altered growth dynamics in response to high ambient temperature involve the thermosensor PhyB and the downstream transcription factor PIF4. The high-temperature signal perceived by PhyB is relayed through PIF4 to auxin biosynthetic and signalling genes, such as *YUCCA8* and *IAA29*, leading to increased auxin response [14–16]. Increased auxin levels trigger a plethora of auxin signalling responses by inducing the expression of *Aux*/*IAA*, *ATHB2*, and *SAUR* genes, leading to growth adjustments that mitigate the effects of temperature fluctuations [17]. Such growth effects are mediated by the regulation of downstream cellular attributes that govern cell proliferation, differentiation, and growth. For instance, adjustment in *Arabidopsis* root growth dynamics at various growth temperatures from 15–30°C is shown to involve both cell elongation and proliferation [18]. The contributions of longer cell length and faster proliferation to root length increase with temperature, suggesting that the adaptive response to temperature involves dynamic regulation of cell proliferation and cell-cycle progression.

Similar to other eukaryotic systems, the *Arabidopsis* cell cycle consists of four major phases: Gap1 (G1), Synthesis (S), Gap2 (G2), and Mitosis (M). During the G1 phase, the cell grows and assesses environmental conditions before entering the S phase [19,20]. The genome is replicated during the S phase, followed by the G2 phase, where cells prepare for mitosis. Chromosomal segregation in the M phase is followed by cytokinesis, resulting in two daughter cells. Cells often enter the endocycle to improve fitness under adverse environmental conditions, a process that involves DNA replication without cell division, leading to increased ploidy levels [21,22]. CYCLINS AND CYCLIN-DEPENDENT KINASES (CDKs), which are regulated by a plethora of internal and external cues, control progression through the cell cycle to ensure developmental plasticity in response to environmental changes. Specific cyclins and CDKs are involved in the progression through different phases of the cell cycle. Cyclin Ds and CDKA;1 primarily regulate the G1/S transition, whereas A and B-type cyclins regulate DNA replication and mitotic progression in *Arabidopsis* [19,23]. The readiness to enter the next phase is assessed by ‘cell-cycle checkpoints’, which coordinate with cellular and environmental conditions to ensure accurate genome replication and division. Any abnormality or deviation from optimal conditions will arrest or stall the cell cycle until it is resolved [19]. Consequently, temperature-mediated regulation of growth dynamics and cell proliferation must ultimately converge on the modulation of cyclins, CDKs, and associated cell-cycle regulators [24,25].

While there is extensive information on the temperature-mediated adaptive growth responses in *Arabidopsis*, a systematic integration of temperature signalling and cell-cycle control to modulate morphological and developmental changes remains limited. Moreover, a comprehensive compilation of the effects of different growth temperatures on cell division and the cell cycle, and how these regulate growth responses, is lacking. In the present review, we provide an overview of the involvement of the cell cycle and the balance between cell division and differentiation in temperature-mediated *Arabidopsis* growth plasticity. We specifically focus on the growth responses to cold/chilling stress (0–15°C), high ambient temperature (27–30°C), and heat stress (32–38°C) and do not discuss extreme temperatures, such as freezing and severe heat stress, that are detrimental to plant survival and typically lead to growth cessation and, often, cell death [6,7]. We explain how temperature fluctuations affect the intricate regulatory mechanisms governing cell-cycle progression in *Arabidopsis*, aiming to bridge the knowledge gap between environmental sensing and cell proliferation dynamics.

## Cold-temperature-mediated control of cell cycle for growth attenuation

**Figure 1:**
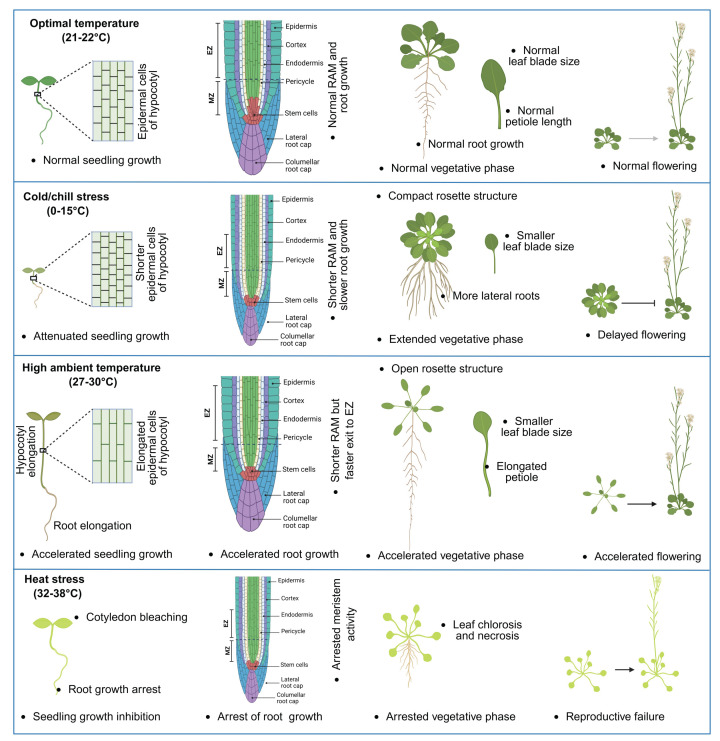
Temperature-dependent regulation of *Arabidopsis* growth dynamics Schematic representation of morphological and developmental responses of *Arabidopsis* exposed to different temperature ranges. Under optimal temperature (21–22°C), seedlings exhibit normal growth and development. Under cold/chilling stress (0–15°C), seedlings display attenuated seedling growth. Root development is characterised by shorter RAM and slower root growth, leading to increased lateral root formation. Leaves exhibit a compact rosette structure, smaller leaves, an extended vegetative phase, and delayed flowering. At high ambient temperature (27–30°C), hypocotyl and root elongation promote accelerated seedling growth. In particular, roots exhibit reduced meristem size but faster exit to the elongation zone, thereby maintaining faster root growth. Plants display an open rosette architecture, elongated petioles, smaller leaf blades, and accelerated flowering. Heat stress (32–38°C) induces severe growth defects by arresting meristem activity. Plants are wilted and often fail to reproduce due to reduced pollen viability and seed production.

**Figure 2:**
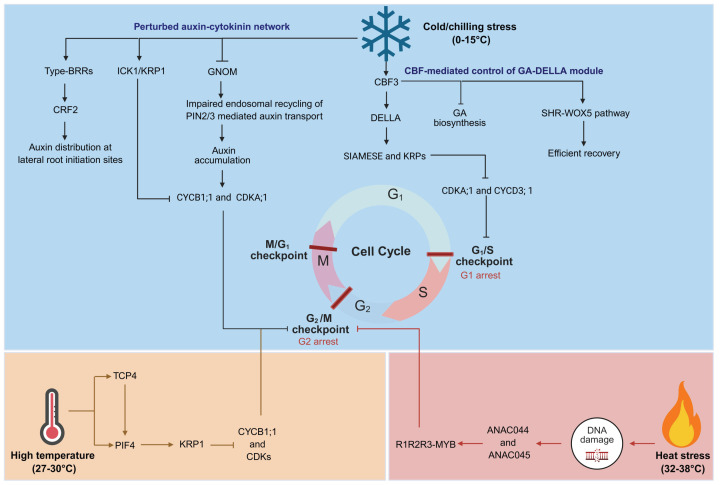
Temperature regulation of cell-cycle dynamics to shape *Arabidopsis* growth Under cold/chilling stress (0–15°C), perturbation of the auxin–cytokinin network alters auxin distribution and transport by regulating Type-B RRs, CRF2, and GNOM-dependent PIN2/3 recycling, leading to auxin accumulation. These changes suppress the activity of core cell-cycle regulators, including CDKA;1 and CYCB1;1. In parallel, CBF-mediated signalling controls the GA–DELLA module, where CBF3 promotes DELLA accumulation and represses GA biosynthesis. This induces SIAMESE and KRP proteins, inhibiting CDKA;1 and CYCD3;1 activity and resulting in G_1_/S cell-cycle arrest. Cold signalling also engages the SHR–WOX5 pathway to support meristem maintenance and recovery. At high ambient temperatures (27–30°C), TCP4 and PIF4 regulators promote the cell-cycle inhibitor KRP1, contributing to reduced cell proliferation and smaller leaf blades. Heat stress (32–35°C) induces DNA damage, leading to the induction of NAC transcription factors ANAC044 and ANAC045, which, in turn, regulate R1R2R3-MYB proteins. These pathways inhibit cell-cycle progression, particularly at the G_2_/M checkpoint, leading to G2-phase arrest. Collectively, temperature-dependent signalling pathways converge on key cell-cycle checkpoints (G_1_/S and G_2_/M) to regulate cell division and plant growth responses under varying temperature conditions.

*Arabidopsis* plants exhibit attenuated shoot growth, reduced primary root growth with increased lateral root density, and delayed reproductive transition under cold/chilling temperatures [26–30]. Rosettes are smaller and have more leaves due to an extended vegetative phase, resulting in a compact structure that aids in insulation at lower temperatures [28,31] (Figure 1). This growth attenuation is primarily mediated by cell-cycle arrest and/or a reduction in cell production rate [32]. Cold temperatures alter meristem organisation, inhibiting *Arabidopsis* root growth. *Arabidopsis* plants grown at low temperature (10°C) display either a similar or slightly smaller root meristem than the plants grown at 21°C [32] (Figure 1). However, the root meristem exposed to low temperature shows increased *CYCB1;1* expression, suggesting that cell-cycle progression might be halted at the G2/M phase. Consistent with this, low temperature reduced the number of cells entering the S phase and led to an accumulation of cells in the G1/G2 phase in maize [33]. Kinematic studies also showed reduced meristem size at low temperature, but with constant cell production rates and altered transit times through the meristem zone [18]. The increased Cyclin B1;1 expression in meristem cells and the altered transit time to exit the meristem demonstrate that low temperatures directly influence cell-cycle progression and meristem organisation. A recent study has investigated the mechanism underlying the low-temperature-mediated ‘brake’ on the cell cycle in *Arabidopsis* roots. Cold stress activates a pause-and-play mechanism that temporarily suppresses mitotic progression at the G2/M transition, which resumes during recovery. This is mediated by the activation of inhibitor of CYCLIN-DEPENDENT KINASE 1/KIP-RELATED PROTEIN 1 (KRP1), which suppresses the activity of CYCB1;1 and associated CDKs [32] (Figure 2). The arrest of the cell-cycle progression serves as a protective mechanism to ensure genomic integrity and to support cellular repair processes before further division, thereby aiding efficient recovery after stress cessation. The SCARECROW (SCR)–SHORTROOT (SHR)–WUSCHEL-RELATED HOMEOBOX 5 (WOX5) pathway is a critical genetic cascade that maintains the stem cell niche and quiescent centre identity in *Arabidopsis* roots [34–38]. Low temperature induces *CBF3* expression in the endodermis, which then migrates to the quiescent centre to maintain the SHR-WOX5 pathway and ensure efficient recovery (Figure 2). The *cbf3* mutant displays impaired root recovery after low temperature exposure [39].

The interaction of temperature signalling components with phytohormone signalling networks is critical for *Arabidopsis* growth dynamics. Cold temperatures strongly affect the auxin-cytokinin regulatory network controlling the root meristem activity [40]. Auxin and cytokinin function antagonistically to balance cell proliferation and differentiation in the *Arabidopsis* root meristem for maintaining meristem activity and cell production [41,42]. Auxin maxima are maintained around the quiescent centre (QC) to maintain stem cell identity in the root apical meristem (RAM). This is primarily mediated by polar auxin transport via PIN FORMED (PINs). In contrast, cytokinin antagonises auxin in the RAM by promoting the degradation of PIN proteins, thereby disrupting polar auxin transport and promoting cell differentiation [43,44]. As PIN proteins are crucial for maintaining auxin maxima, they are constantly recycled and re-established by clathrin-mediated endocytosis via the GNOM pathway. They are endocytosed from the plasma membrane into the *trans*-Golgi network/early endosome and recycled back to the plasma membrane or targeted for vacuolar degradation [45]. Cold stress impairs endosomal recycling of PIN2/3-mediated auxin transport by reducing *GNOM* expression, thereby affecting vesicle trafficking and PIN recycling [29,33]. Hence, polar auxin transport is disrupted at low temperatures, leading to auxin accumulation in RAM. The accumulated auxin represses CYCB1;1 and CDKA;1 and inhibits the G2-M transition, leading to an extended G phase and shortened meristems (Figure 2). Moreover, cold temperatures integrate cytokinin signalling by inducing type B response regulators (RRs), thereby increasing transcription of *CYTOKININ RESPONSE FACTOR 2* (*CRF2*). The cold-induced CRF2 activity promotes PIN-mediated auxin distribution at lateral root initiation sites, thereby facilitating lateral root formation [33,46]. In addition, cold-temperature-mediated control of root growth involves the gibberellin (GA) signalling pathway. Low temperatures reduce GA biosynthesis and accumulation via the CBF signalling pathway. Therefore, DELLA proteins, negative regulators of GA signalling, might contribute to the observed cell-cycle inhibition under low temperature [47]. DELLAs are known to directly repress cell division by enhancing the expression of cell-cycle inhibitors such as SIAMESE and KRPs, which block the activity of CDKA;1 and CYCD3;1 and arrest the cell cycle at the G1/S phase, leading to shorter meristems [19] (Figure 2). Consistent with this, *della* mutants retain root elongation at low temperatures, indicating that critical functions of these proteins are involved in low-temperature-mediated alterations in cell-cycle progression [48]. The recovery of RAM upon return to normal temperature requires the rebalancing of the auxin maxima. A survival-by-sacrifice mechanism operates in the RAM, where newly generated columella stem cell daughters (CSCDs) are preferentially lost, thereby protecting the QC. This is mediated by the DNA damage response proteins ATR, ATM, and WEE1, as their mutants failed to selectively sacrifice CSCDs and thus increased the vulnerability of QC and the entire stem cell niche [49].

In summary, the cold-induced attenuation of *Arabidopsis* growth involves regulation of the cell cycle, either through hormonal signalling or direct checkpoint control [6]. The coordinated suppression of auxin transport, through compromised GNOM-mediated trafficking, and the reduction in GA signalling, facilitate cell-cycle inhibitor, which collectively enforce the mitotic arrest [50]. This mitotic arrest, in addition to serving as a growth restraint, also functions as a critical adaptive strategy to protect genomic integrity and preserve the meristem’s potential for efficient recovery once favourable conditions return [49].

## Cell-cycle regulation for growth dynamics under high ambient temperature

*Arabidopsis* growth responses at high ambient temperatures, termed thermomorphogenesis, involve hypocotyl and petiole elongation, hyponastic growth, and reduced leaf area, resulting in an open rosette structure to facilitate cooling [51–53] (Figure 1). These phenotypic changes associated with thermomorphogenesis are often driven by accelerated cell proliferation and expansion, alongside altered cell cycle progression, to facilitate rapid growth [18,54]. Higher temperatures promote cell division and elongation in roots, enabling deeper growth into the soil and seeking out better moisture [52–55] (Figure 1). High ambient temperature promotes growth not merely by increasing cell production but by coordinating adjustments in the dynamics of proliferation and elongation in roots. Although root elongation occurs at high temperatures, the overall cell production rate and elongation zone length do not change significantly. Nonetheless, root growth is maintained by reduced meristem size, a faster exit from the meristem into the elongation zone, and a compensatory increase in cell elongation rates. Thus, the high-temperature-dependent growth responses in the root meristem result from a redistribution of developmental timing, rather than a simple increase in cell division [18]. Consistent with this, a recent study with 34 *Arabidopsis* accessions and mutants showed that cell division is highly plastic and can be compensated for by altering developmental timing and elongation rates, suggesting that cell division rates are not rigid determinants of root growth [56]. Despite studies emphasising a balance between cell proliferation and elongation in root thermomorphogenesis, a report indicates that root growth promotion in response to high temperatures is primarily due to increased cell division rates in the root apical meristem [54]. Higher temperatures increase auxin biosynthesis in roots and elevate *PIN1/2* expression, thereby enhancing auxin levels in the root tip, which increases cell division rates and promotes primary root elongation [54]. In addition to increased auxin production, high temperature also influences auxin homeostasis by enhancing the SORTING NEXIN1, a nexin protein involved in endosomal membrane trafficking, which recycles and maintains the plasma membrane localisation of PIN2 [57]. This homeostasis might influence the balance between cell division and differentiation. In contrast with auxin accumulation, prolonged high temperature decreases brassinosteroid signalling by reducing the abundance of BRI1, a brassinosteroid receptor. This leads to reduced meristem size due to the early departure of cells from the proliferative to the elongation zone [58]. Despite differences in the reports, which suggest either a primary role of cell division or a balance between cell proliferation and elongation in root thermomorphogenesis, the studies underscore the highly sensitive and plastic response of the root meristem to high temperature.

*Arabidopsis* shoot thermomorphogenesis is typically characterised by hypocotyl and petiole elongation, as well as reduced leaf area [52] (Figure 1). While high-temperature-mediated elongation response primarily involves cell elongation, leaf growth plasticity under high ambient temperature results from direct regulation of the cell cycle via temperature-responsive transcription factors. High ambient temperatures inhibit cell proliferation, reducing leaf area. PIF4, a central regulator of thermomorphogenesis, interacts with the transcription factor TEOSINTE BRANCHED1/CYCLOIDEA/PCF4 (TCP4) to activate expression of the cell-cycle inhibitor KRP1 in proliferating young leaf primordia, resulting in reduced cell division and smaller leaves [59] (Figures 1 and 2). Interestingly, temperature is also critical for proper stem cell maintenance in *Arabidopsis* shoot apical meristems. As the WUSCHEL (WUS)–CLAVATA (CLV) pathway ensures proper stem cell maintenance, the *clv* mutant shows defects in stem cells, leading to fasciated meristems and termination of leaf and floral primordia. However, high-temperature-mediated increased auxin biosynthesis partially rescues this impaired stem cell regulation in *clv*, suggesting that thermomorphogenesis can compensate for the WUS–CLV pathway [60].

Unlike cold, high ambient temperature shows a distinct spatial pattern of cell-cycle regulation, where proliferation is promoted in roots and inhibited in leaves. This, while highlighting the sophistication of plant temperature response and the plasticity of the cell cycle, also showcases the remarkable ability of plants to remodel their growth dynamics and architecture to survive temperature changes.

## Heat stress adversely affects the cell cycle

Heat stress (32–38°C) induces DNA damage, protein misfolding, membrane leakage, and ROS accumulation, leading to irreversible oxidative damage [61–63]. As a result, *Arabidopsis* plants exhibit severe growth attenuation, wilting, reduced pollen viability, reduced fertility, and reduced seed set under heat stress (Figure 1). Heat stress inhibits mitotic progression by suppressing the expression of cyclins and CDKs, disrupting microtubule organisation and chromosome segregation, inducing DNA damage in proliferating cells, and further contributing to mitotic arrest and reduced meristematic proliferation [64]. *Arabidopsis* root growth attenuation under heat stress (32°C) is attributed to reduced RAM size and fewer cells expressing *CYCB1;1*. This reduced RAM size was further complemented by more cells expressing WOX5-GFP in heat than in control, likely compensating for the premature cessation of cell proliferation or heat-stress-induced cell damage. The stem cell damage response transcription factor ERF115 is up-regulated by heat stress. The *erf115* mutant plants exhibit more pronounced attenuation of root length and reduced meristem size compared with the control, reinforcing their role in stem cell replenishment and cell division [64,65]. Similarly, the NAC transcription factors ANAC044 and ANAC045, which are involved in stem cell death, are induced in response to DNA damage caused by heat stress in *Arabidopsis* roots. These transcription factors facilitate the accumulation of R1R2R3-MYB transcription factors, which arrest progression from G2 to M phase (Figure 2). Mutants of these ANAC transcription factors are thermotolerant, and heat-stress-mediated growth arrest requires this module. Heat-stress-mediated induction of ANAC044 and ANAC045 is also hypothesised to serve as a checkpoint to determine cell fate under heat stress, whether to enter programmed cell death or undergo endoreduplication [20]. In addition to mitotic cell cycle effects, heat stress significantly affects meiotic cell division by inducing premature separation of sister chromatids before anaphase II, leading to disrupted cohesion between sister centromeres. Moreover, heat stress affects cytokinesis by disrupting the phragmoplast or microtubular organisation [66]. Heat induces the production of 2n gametes, termed heat-induced meiotic restitution, in which the first or second meiotic division is skipped. Recently, the core meiotic cyclin Tardy asynchronous meiosis (CYCA1;2) and three division mutant 1 were identified as regulators that maintain the translation of key cell cycle genes in meiosis. This is required to prevent premature exit from meiosis during heat stress [67]. Together, heat stress primarily attenuates growth, allowing DNA damage repair and cellular stress responses by inhibiting cell proliferation. Hence, while high ambient temperature promotes adaptive developmental reprogramming, heat stress reallocates energy from growth to cellular protection.

## Transcriptomic evidence and future omics-mediated approaches for investigating temperature-mediated cell-cycle regulation

Bulk transcriptomics has provided extensive information on plant temperature responses and major regulators, including the cell-cycle-mediated responses. Extensive transcriptomic information at multiple spatio-temporal resolutions already exists across a range of growth temperatures. Meta-analysis of several transcriptomes from 139 studies across different growth temperatures and tissues identified gene clusters with distinct transcription patterns: some specific to certain temperatures, such as CBFs in cold, and others common across different temperatures, such as HEAT SHOCK FACTORS (HSFs) [41]. Several major regulators of temperature response, including CBFs, PIF4, and HSFA1a, were identified as highly variable genes because they exhibited distinct transcriptional profiles across different temperatures and tissues. Many of these transcription factor pathways are well known to integrate with cell-cycle regulatory networks.

While conventional bulk transcriptomic studies have been invaluable for delineating temperature response pathways and regulators, the inherent disadvantage of data derived from heterogeneous cell populations limits the resolution of response pathways at the cellular level. Considering that the meristem comprises a relatively small number of specialised meristematic cells, where the majority of cell cellcycle regulation occurs, the other differentiated cells crowd out meristematic cells, resulting in under-representation of cell-cycle regulators in bulk transcriptomics. The emergence of single-cell/single-nuclei sequencing technologies over the past few years offers an exciting platform for resolving cell-cycle dynamics and their differential regulation across temperatures with unprecedented resolution. Single-cell RNA sequencing (scRNA-seq) of *Arabidopsis* roots has enabled the mapping of distinct cell populations and developmental trajectories for cells progressing from meristematic to mature stages [68,69]. Application of the scRNA-seq approach across different temperature conditions would detect temperature-induced transcriptional changes at cell-type-specific resolution. This would further allow us to distinguish the effects of temperature-mediated transcriptional changes across different cell states, whether in a dividing or differentiating stage, clarifying the reported dynamics of dividing versus expanding tissues. In addition, pseudotime and trajectory analyses facilitated by scRNA-seq will provide insight into the effects of temperature on the dynamics of developmental and cell-cycle progression [70]. The single-cell sequencing strategy will also help profile differences in cell proportions at different phases of the cell cycle, whether the number of dividing cells is reduced, cells are arrested at a certain stage, etc. Moreover, the spatial transcriptomics techniques will help map expression changes to precise locations, providing spatial context for gene expression and thus enabling precise mapping of transcriptional changes to developmental zones [71,72]. The latest multi-omics platforms enable profiling of transcriptomic changes alongside chromatin accessibility changes at cellular resolution by combining RNA-seq with single-nucleus ATAC sequencing from the same nucleus [73,74]. This aids in identifying cell-type-specific regulatory elements and transcription factor-binding landscapes, along with chromatin accessibility, that underlie temperature-induced changes in gene expression. In addition to chromatin accessibility, the emerging single-cell epigenomic technologies facilitate the investigation of chromatin and epigenetic modifications as additional regulatory layers. For example, single-nucleus cleavage under targets and tagmentation can profile histone modifications, while single-nucleus DNA methylome profiling can assess DNA methylation changes at the cellular level and chromatin conformation [75]. Together, these strategies would not only enhance our understanding of the temperature regulation of cell-cycle dynamics at spatial and temporal resolutions but also provide unprecedented resolution into temperature-induced changes in gene expression across distinct cell types and the coordination among the different regulatory layers at the cellular level.

## Conclusion

Plant growth and development are coordinated by cell-cycle dynamics that involve cell progression and fate. The developmental and environmental signals converge on the cell-cycle dynamics to mediate plant adaptive responses in terms of growth plasticity. Hence, a comprehensive understanding of the temperature-dependent regulation of the cell-cycle is of prime importance for optimising plant growth responses to temperature fluctuations. Despite having an overview of plant growth responses to different temperature regimes, the integration of temperature cues with cell-cycle regulators and dynamics is limited. Here, we outline the distinct effects of different temperature ranges on cell-cycle dynamics across *Arabidopsis* organs, particularly leaves and roots. Roots, due to their relative ease of imaging and visualisation, are extensively studied for cell cycle and division dynamics, whereas shoot meristems and leaves are less studied. Nonetheless, the leaf shows remarkable growth plasticity in response to temperature changes due to its developmental complexity. Therefore, coordination of temperature signals with several novel regulators and cell-cycle components is expected to drive leaf growth dynamics [59]. While moderate temperature changes generally alter cell-cycle dynamics to manifest developmental plasticity, extreme temperature conditions often arrest cell-cycle progression. Temperature regulation of cell-cycle dynamics for growth plasticity requires integration of temperature-responsive regulators with the core cell-cycle components. For example, the cold-mediated regulation of the cell cycle involves the conventional cold response pathway, CBF-COR [39], while the major high-temperature regulator, PIF4, regulates *KRP* expression to mediate high-temperature-induced reduction in cell proliferation in leaves [59]. While upstream temperature perception and signalling have been investigated in detail, the knowledge of their convergence in the cell cycle remains limited. Recent advances in live-cell imaging, cell-cycle reporter systems, and single-cell sequencing technologies offer an exciting avenue for detailed integration and elucidation of cell-cycle dynamics in response to temperature changes. For example, the multi-cell-cycle reporter system placci offers an opportunity to track cell-cycle progression in response to temperature dynamics [76]. Single-cell sequencing technologies will help decipher the molecular signals governing the cell-cycle phase transition in response to temperature [77]. Similarly, advanced live-cell imaging will expedite the investigation of cell-cycle dynamics in the shoot apical meristem and leaf under temperature changes. Investigating the epigenetic regulation of the cell-cycle dynamics in response to temperature changes is another exciting avenue, as the importance of epigenetic and chromatin changes in temperature-mediated adaptive responses is well documented [78].

Future studies integrating live-cell imaging, cell-cycle reporter systems, single-cell multi-omics, and chromatin dynamics will be instrumental in dissecting the influence of temperature on cell proliferation at spatio-temporal resolution. Such studies will provide finer details on meristem behaviour, division kinetics, and cell-cycle progression across different developmental stages and tissues, thereby improving our understanding of plant growth plasticity during thermal adaptation and helping optimise plant developmental responses to temperature fluctuations.

## Perspectives

- With global climate change and temperature fluctuations, understanding plant adaptive responses and their underlying mechanisms is crucial for crop improvement strategies. Temperature significantly affects biomass and fertility, which are primarily orchestrated by the changes in growth dynamics and are regulated by the cell cycle.
- We have highlighted here that major temperature-responsive pathways, including PhyB-PIF, CBF-COR, and HSFs, connect to cell-cycle regulation to mediate adaptive responses, either acclimation or tolerance. However, significant gaps remain regarding how multiple temperature-signalling mechanisms converge at the cell cycle and how they are regulated.
- Extensive spatio-temporal profiling of the cell cycle across different temperatures using various strategies, such as live-cell imaging and cell-cycle marker systems, will provide a better understanding of cell-cycle progression. Further integrating this knowledge with advanced technologies, such as scRNA-seq, will revolutionise our understanding of cell-cycle dynamics under temperature fluctuations, enabling the development of novel strategies for crop thermal adaptation and tolerance.

## Open Access

Open access for this article is provided via our Subscribe to Open programme, made possible through the support of our subscribing institutions. Learn more here: https://portlandpress.com/pages/open_access_options_and_prices#conditional.

## Abbreviations

CBF: C-repeat binding factor
CORs: Cold-responsive genes
CSCDs: Columella stem cell daughters
CRF2: Cytokinin response factor 2
CDKs: Cyclin-dependent kinases
GA: Gibberellin
G1: Gap1
G2: Gap2
HSFs: Heat shock factors
ICE1: Inducer of CBF expression 1
KRP1: Kip-related protein 1
M: Mitosis
PhyB: Phytochrome B
PIF4: Phytochrome-interacting factor 4
PINs: PIN FORMED
QC: Quiescent centre
RAM: Root apical meristem
RRs: Response regulators
SCR: SCARECROW
scRNA-seq: Single-cell RNA sequencing
SHR: SHORTROOT
TCP4: TEOSINTE BRANCHED1/CYCLOIDEA/PCF4
CLV: CLAVATA
WUS: WUSCHEL
WOX5: WUSCHEL-RELATED HOMEOBOX 5
